# Supplementary material for: Common mouse models of chronic kidney disease are not associated with cachexia
Source: Commun Biol. 2024 Mar 20;7:346. doi: 10.1038/s42003-024-06021-y (PMC10954638; doi:10.1038/s42003-024-06021-y)
Supplement: Supplementary file 2 — Supplementary Information [file 42003_2024_6021_MOESM2_ESM.pdf]

Common mouse models of chronic kidney disease are not associated with cachexia

Benjamin Lair[1], Marlène Lac[1], Lucas Frassin[1], Manon Brunet[2], Marie Buléon[2], Guylène Feuillet[2], Claire Maslo[1], Marie Marquès[1], Laurent Monbrun[1], Virginie Bourlier[1], Emilie Montastier[1], Nathalie Viguerie[1], Geneviève Tavernier[1], Claire Laurens[1], and Cedric Moro[1] *

Supplementary Table 1. List of primer sequences used for real-time qPCR with SYBR chemistry:

Gene symbol	Forward	Revers<br>e
Fbxo32	ACGTAGTAAGGCTGTTGGAGC	GTTCTTTTGGGCGATGCCAC
Trim63	TGCCTGGAGATGTTTACCAAGC	AAACGACCTCCAGACATGGACA
Mstn	AGATGGGCTGAATCCCTTTTT	GCAGTCAAGCCCAAAGTCTC
Tbp	GGAATTGTACCGCAGCTTCAAA	GATGACTGCAGCAAATCGCTT

Supplementary Table 2. List of Taqman probes used for real-time qPCR:

**Gene symbol**	**Taqman Probe ID**
Tnfa	Mm00443258_m1
Il6	Mm00446190_m1
Il1b	Mm01336189_m1
Ccl2	Mm00441242_m1

Supplementary Figure Legends

Supplementary Figure 1. 5/6 nephrectomy reduces adipose tissue mass. Plasma urea, plasma (**a**) creatinine (**b**), and body weight (**c**) in sham (black bars) versus Nx (red bars) mice 12 weeks after the first surgery (PS1). Mass of inguinal white adipose tissue (iWAT) and epididymal adipose tissue (eWAT) from Sham and Nx mice 12 weeks after the first surgery (n=10 mice per group) (**d**). Respiratory exchange ratio (RER) time-course over 24h as measured in calorimetric chamber 6 weeks after the first surgery (**e**), mean RER (**f**) and locomotor activity (**g**) in Sham (black bars/lines) versus Nx (red bars/lines) mice (n=8 mice per group). Plasma triglyceride (TG) (**h**) and Free fatty acids (FFA) (**i**) in Sham and Nx mice 6 weeks after the first surgery (n=8-10 mice per group). Data are expressed as means ± s.e.m. Statistical significance was calculated by unpaired t-tests and two-way ANOVA accordingly. *** $p<0.001$, **** $p<0.0001$.

Supplementary Figure 2. 5/6 nephrectomy does not affect muscle phenotype or inflammation profile. Relative quantification of atrogene (**a**) and pro-inflammatory (**b**) mRNA in the *gastrocnemius* muscle of Sham (black bars) versus Nx (red bars) mice, 6 weeks after the first surgery (n=6 mice per group). Relative quantification of atrogene (**c**) and pro-inflammatory (**d**) mRNA in the *Soleus* muscle of Sham versus Nx mice, 6 weeks after the first surgery (n=6-8 mice per group) Plasma concentrations of TNFα, IL6, IL1β and CCL2, in sham versus Nx mice, 6 weeks PS1 (**e**) (n=6 mice per group). Data are expressed as means ± s.e.m. Statistical significance was calculated by multiple unpaired t-tests with Holm-Šídák's multiple comparisons. * $p<0.05$, **** $p<0.0001$.

Supplementary Figure 3. Preserved energy metabolism after adenine diet removal. Time-course of cumulated food intake over 24h (**a**) and average 24h food intake (**b**) measured in

metabolic cages in control (black bars/lines) and adenine (orange bars/lines) mice 3 weeks after switching the mice back to standard diet (n=8 mice per group). Time-course of energy expenditure over 24h measured (**c**) and average exergy expenditure (**d**) measured in metabolic cages in control (black bars/lines) and adenine (orange bars/lines) mice 3 weeks after switching the mice back to standard diet (n=8 mice per group). Water intake (**e**) and locomotor activity (**f**) measured over 24h in metabolic cages 3 weeks after switching the mice back to standard diet (n=8 mice per group). Data are expressed as means ± s.e.m. Statistical significance was calculated by unpaired t-tests and two-way ANOVA accordingly. *** $p<0.001$.

Supplementary Figure 4. Changes in body composition in the adenine diet model. Longitudinal changes of lean mass (**a**), final lean mass (**b**), and lean mass gain (**c**) of control (black bars/lines) versus adenine (orange bars/lines) mice during the first 5 weeks following adenine diet introduction. Longitudinal changes of fat mass (**d**), final fat mass (**e**), and fat mass gain (**f**) of control (black bars/lines) versus adenine (orange bars/lines) mice during the first 5 weeks following adenine diet introduction. Final body weight (**g**) and body weight gain (h) after reintroduction of the standard diet. Mass of iWAT and eWAT adipose tissue depots (**i**) from control and adenine mice at the end of the protocol (9 weeks) (n=10 mice per group). Data are expressed as means ± s.e.m. Statistical significance was calculated by unpaired t-tests and two-way ANOVA accordingly. * $p<0.05$, ** $p<0.01$, *** $p<0.001$, **** $p<0.0001$.

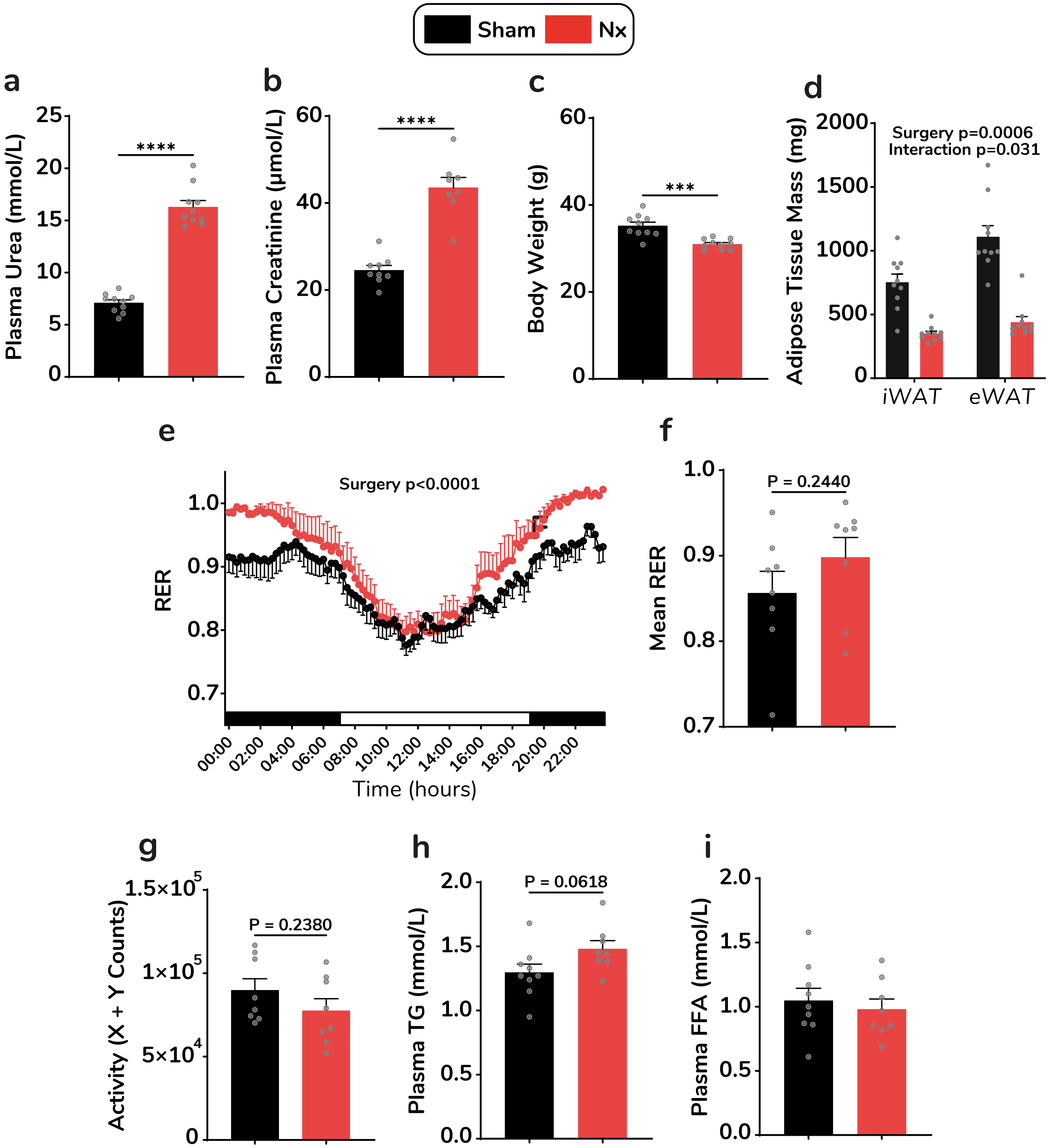

Supplementary Figure 1

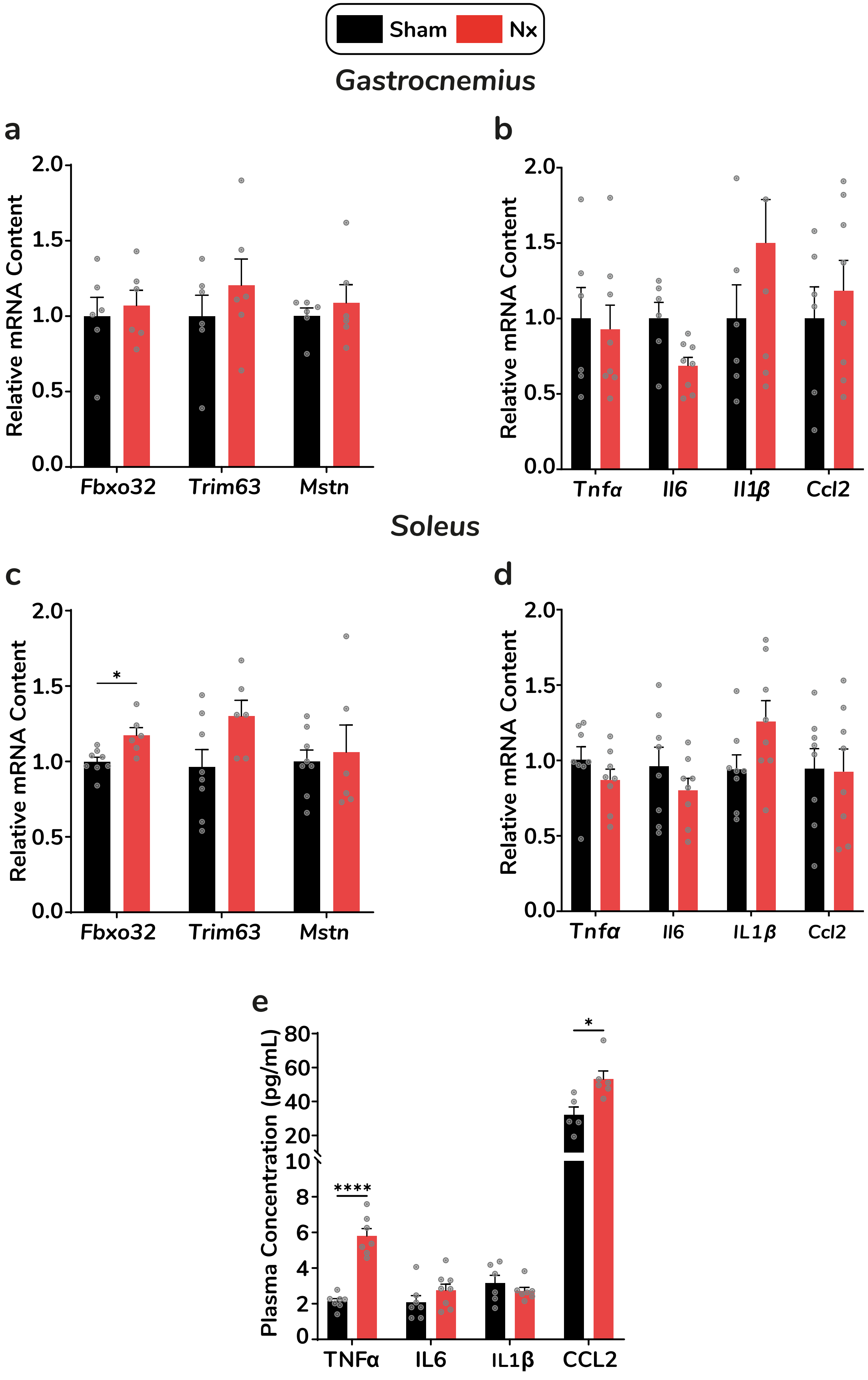

Supplementary Figure 2

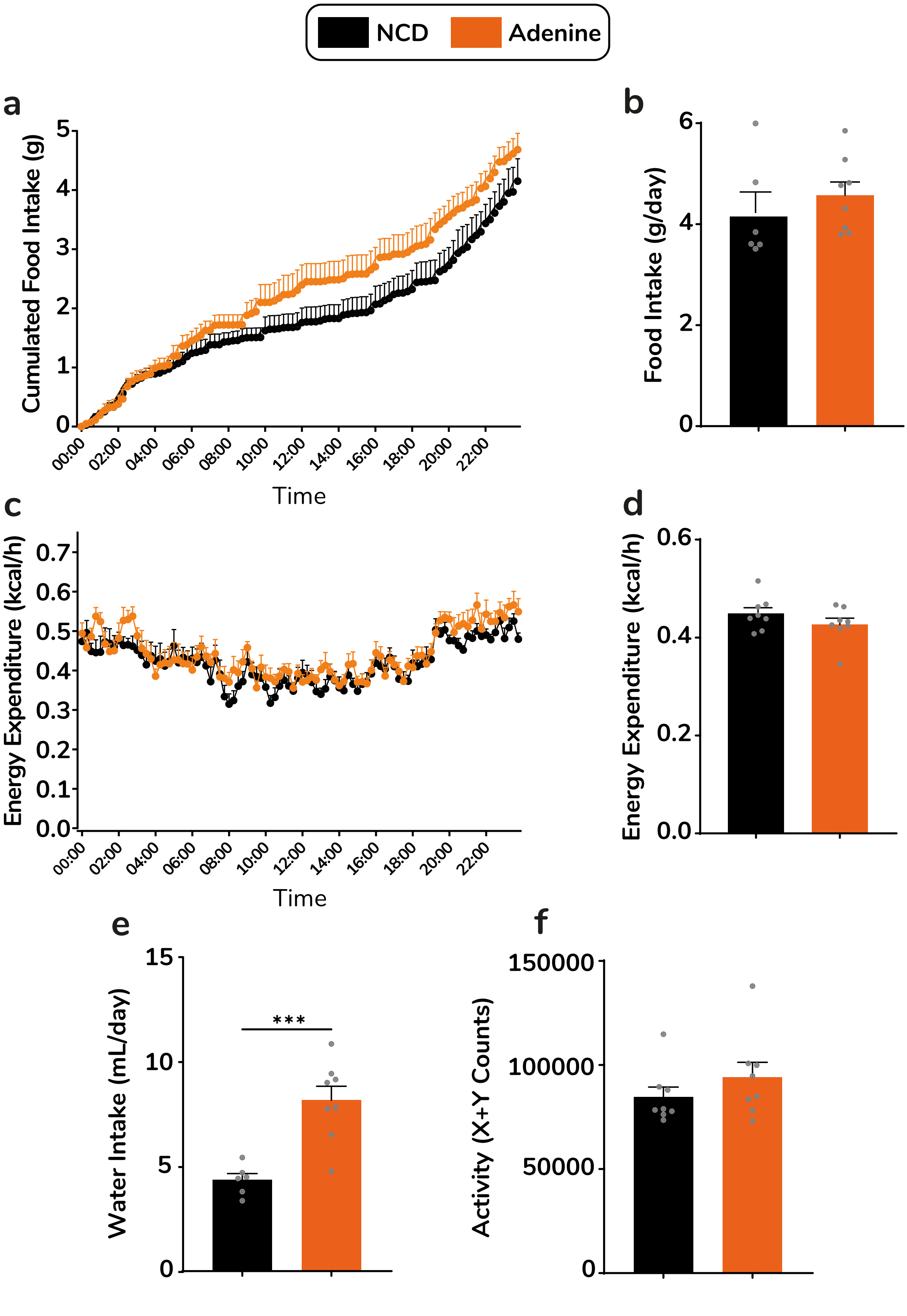

Supplementary Figure 3

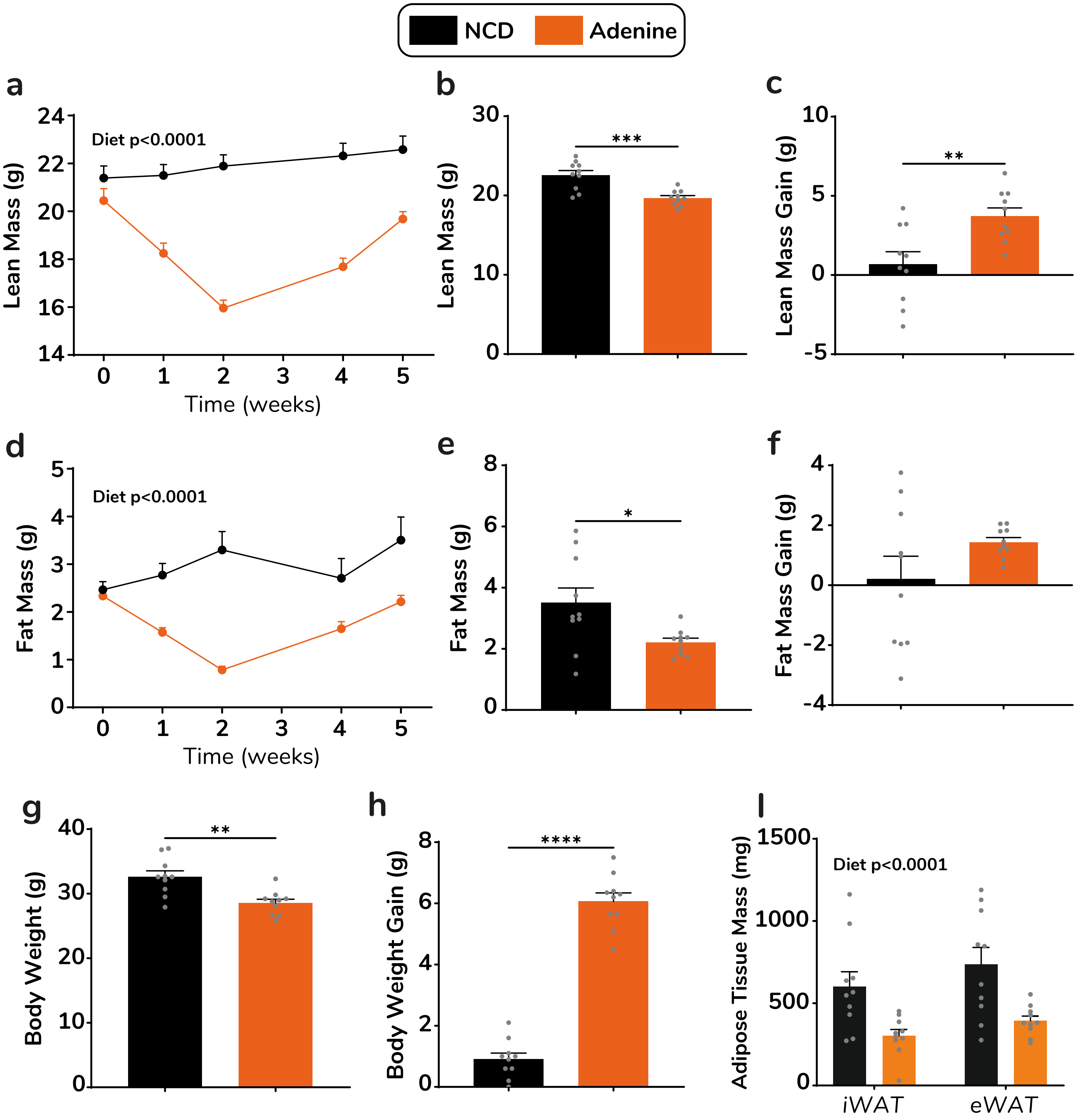

Supplementary Figure 4
